# Identification of Key Genes in Acute Liver Failure via Analysis of Programmed Cell Death Patterns

**DOI:** 10.1155/ijog/4119010

**Published:** 2026-03-19

**Authors:** Guangli Liu, Huili Wu, Wenxiang Shi, Chenjie Qiu

**Affiliations:** ^1^ Department of General Surgery, Changzhou Hospital of Traditional Chinese Medicine, Changzhou, China, czzyy.com; ^2^ Department of Endodontics, Changzhou Hospital of Traditional Chinese Medicine, Changzhou, China, czzyy.com; ^3^ Department of Pediatric Cardiology, Xinhua Hospital, Affiliated to Shanghai Jiao Tong University School of Medicine, Shanghai, China

**Keywords:** bioinformatics, HBV–ALF, immune infiltration, machine learning, programmed cell death

## Abstract

**Objective:**

Acute liver failure (ALF) is a severe condition with high mortality, where programmed cell death (PCD) plays a critical yet not fully understood role. This study aimed to identify key PCD‐related genes and explore their potential as biomarkers and therapeutic targets in ALF.

**Methods:**

Three HBV–ALF microarray datasets (GSE14668, GSE38941, GSE62029) from GEO were integrated and analyzed. Differential expression analysis, protein–protein interaction (PPI) network construction, and functional enrichment were performed. Machine learning algorithms (LASSO and random forest) were used to identify hub genes. Immune infiltration was assessed via CIBERSORT and ssGSEA. Regulatory networks involving miRNAs and transcription factors (TFs) were constructed.

**Results:**

A total of 109 differentially expressed PCD genes were identified. TIMP1 and IL18 were consistently selected as hub genes by both CytoHubba and machine learning methods. These genes demonstrated high diagnostic accuracy and prognostic value in ALF. Immune infiltration analysis revealed significant associations with macrophage polarization. Functional enrichment linked TIMP1 and IL18 to immune and metabolic pathways. A miRNA–mRNA–TF regulatory network was constructed, with STAT3 identified as a key upstream regulator.

**Conclusion:**

TIMP1 and IL18 are potential diagnostic biomarkers and candidate therapeutic targets for ALF, closely associated with immune infiltration and PCD processes. These findings provide new insights into the molecular mechanisms of ALF and support the development of targeted therapies.

## 1. Introduction

Acute liver failure (ALF) is characterized by an abrupt onset of extensive hepatocyte necrosis within a brief timeframe. This condition results in a severe impairment of liver functions, including synthesis, detoxification, excretion, and biological transformation, manifesting clinically with coagulopathy, jaundice, hepatic encephalopathy, and ascites, among other prominent features [1, 2]. A variety of factors can precipitate ALF, encompassing acetaminophen toxicity, hepatic ischemia, viral and autoimmune hepatitis, and drug‐induced liver injury stemming from prescription drugs, herbal remedies, and dietary supplements [3]. Notably, acetaminophen stands out as the predominant contributor to ALF in North America (45.7%) and the United Kingdom (65.4%), while viral infections, particularly Hepatitis B virus (HBV), are the primary etiological factors in Asia and Africa [4–6]. The underlying pathological mechanisms of ALF encompass direct injury to both parenchymal and nonparenchymal liver cells induced by viruses or drugs, immune‐mediated damage, inflammatory responses, and ischemic/hypoxic injuries that exacerbate hepatic insult, culminating in endotoxemia. ALF carries a grim prognosis, with an associated mortality rate ranging between 30% and 50% in the absence of timely intervention [3]. Liver transplantation presently stands as the most effective therapeutic recourse for end‐stage ALF, significantly lowering mortality rates to 20% [7]. However, challenges such as donor scarcity, exorbitant surgical costs, and postoperative immune rejection substantially curtail its widespread applicability. Consequently, the identification of novel therapeutic targets for ALF emerges as a pressing global scientific imperative.

In cases of severe damage where the reparative response proves inadequate for protection or recovery, cell death may ensue, comprising both programmed cell death (PCD) and accidental cell death [8]. Over the past decade, beyond the well‐established modes of cell death (necrosis and apoptosis), a plethora of novel cell death mechanisms have been unearthed and verified, with multiple instances of PCD identified in ALF. For instance, Zhou et al. demonstrated that GDF11 inhibited hepatocyte proliferation and promoted hepatocyte apoptosis via the PI3K/Akt/mTOR signaling pathway, precipitating ALF [9]. Li et al. observed diminished G3BP1 expression during ALF development, linking reduced G3BP1 to the facilitation of P53 intranuclear transfer, promotion of SLC7A11 expression, and induction of ferroptosis [10]. Conversely, Tao et al. identified heightened expression of GAS1 in ALF, which exerted an opposing effect to G3BP1 by fostering ferroptosis and triggering ALF [11]. In a murine ALF model, excessive pyroptosis was identified as a contributory factor to ALF development [12], with subsequent studies affirming that inhibiting pyroptosis effectively mitigated ALF [13]. Additionally, Zhang et al. established that TDZD‐8, an inhibitor of GSK3β, significantly mitigated ALF progression. Suppression of GSK3β activity not only diminished hepatocyte apoptosis, pyroptosis, and necroptosis but also ameliorated liver dysfunction and tissue damage [14]. Furthermore, autophagy and necroptosis were implicated in ALF, with RNF115 inactivation identified as a potential avenue for ALF improvement by promoting autophagy and curtailing the inflammatory response in mice [15]. Elevated RIPK3, a marker of necroptosis, was notably correlated with ALF severity, suggesting its potential utility as a prognostic marker [16, 17].

While various PCD patterns play a crucial role in ALF, our comprehension of the intricate relationship between PCD and ALF remains incomplete. Given the multitude of molecules governing PCD regulation, leveraging public databases becomes paramount in identifying key molecules associated with ALF. This study endeavors to employ bioinformatics and machine learning methodologies to sift through pivotal PCD molecules, construct a predictive nomogram for assessing ALF risk, and scrutinize the correlation between hub PCD genes and immune cells, biological functions, and upstream regulators, among other factors, to offer avenues for precise treatment. The findings of this study lay a foundation for unearthing novel targets in the realm of ALF development.

## 2. Materials and Methods

### 2.1. Data Sources and Processing

Three microarray datasets (GSE14668, GSE38941, and GSE62029) pertaining to HBV‐associated ALF were retrieved from the GEO database (https://www.ncbi.nlm.nih.gov/geo/), all of which were constructed on the GPL570 platform. The study encompassed a total of 85 tissue samples, comprising 47 normal liver tissues and 38 ALF tissues. Additionally, the datasets GSE120652 and GSE74000, comprising 5 normal liver tissues and 6 acetaminophen‐induced ALF tissues, were utilized for the validation of hub gene expression levels. To assess the prognostic significance of hub genes, the GSE168048 dataset, comprising 8 patients with survival outcomes and 8 patients who succumbed within 28 days from HBV‐related acute‐on‐chronic liver failure (ACLF), was incorporated. A detailed summary of the six aforementioned datasets is presented in Table 1. To enhance data consistency and reliability, we transformed probes into gene symbols based on platform information and applied normalization to the raw data using the “limma” package. Further, data integration was executed using the “sva” package to augment the sample size. We used the default parameters for combat correction and used “dataset source” as a batch covariate for correction. Diverse PCD genes were identified by drawing upon information from previously published studies, encapsulating 15 distinct types of cell death [18].

**TABLE 1:** Overview of GEO datasets.

ID	Platform	Species	Samples
GSE14668	GPL570	*Homo sapiens*	20 Normal + 8 HBV–ALF
GSE38941	GPL570	*Homo sapiens*	10 Normal + 17 HBV–ALF
GSE62029	GPL570	*Homo sapiens*	17 Normal + 13 HBV–ALF
GSE120652	GPL6244	*Homo sapiens*	3 Normal + 3 APAP–ALF
GSE74000	GPL570	*Homo sapiens*	2 Normal + 3 APAP–ALF
GSE168048	GPL21185	*Homo sapiens*	8 Survival + 8 Death HBV–ACLF

### 2.2. Differentially Expressed Analysis

We employed two primary methods for the identification of differentially expressed genes (DEGs). The initial method utilized the robust rank aggregation (RRA) approach. Specifically, the three datasets under investigation underwent differential analysis between normal and ALF tissues using the “limma” package. The results of these analyses were subsequently organized using the “RobustRankAggreg” package to derive a set of stable DEGs [19]. The second approach involved conducting a differential analysis on the amalgamated dataset through the “limma” package. Criteria for selection in the aforementioned differential analysis were set at an adjusted *p* value < 0.05 and |log_2_FC| > 2. The DEGs obtained through these two methods were then subjected to intersection to derive the definitive set of DEGs. We adopted two differential expression analysis strategies to enhance the robustness of the results. The RRA method aims to integrate the statistical evidence of multiple independent datasets, reduce the bias of a single dataset, and obtain a more reliable list of differential genes. The batch‐diff limma method makes full use of the increased sample size after integration to improve the statistical test efficiency.

### 2.3. Protein–Protein Interaction (PPI) Network Construction and Module Identification

The DEGs obtained were input into the STRING online tool (https://cn.string-db.org/) for the construction of a PPI network. Subsequently, the Cytoscape software facilitated the visualization and analysis of the PPI network. This encompassed the identification of significant modules using the MCODE plug‐in and the determination of the hub network through the CytoHubba plug‐in. The CytoHubba plug‐in employed ten algorithms (MCC, DMNC, MNC, Degree, BottleNeck, Eccentricity, Closeness, Radiality, Betweenness, and Stress) to assess the importance of genes. We employed these ten distinct algorithms to compute the importance ranking of each gene. For each algorithm, the top 70 genes were selected. The intersection of these ten gene lists was then taken, yielding core candidate genes consistently identified as highly significant across all algorithms.

### 2.4. Identification of Hub Genes Using Machine Learning Algorithm

We used the entire integrated training set (85 samples after the GSE14668+ GSE38941+ GSE62029 combination) to identify the hub genes. While the validation set is completely independent (11 samples from the combined GSE120652 and GSE74000 datasets), there is no sample overlap. Two primary machine learning methodologies were employed to identify potential diagnostic markers for ALF. Initially, based on the classification of the samples, the 17 hub network genes were subjected to least absolute shrinkage and selection operator (LASSO) analysis using the “glmnet” package and ten‐fold cross‐validation. Variables with the minimum log lambda were deemed characteristic variables so that the model has better generalization ability on the test set to minimize the risk of overfitting. Additionally, random forest (RF) analysis with parameters set as ntree = 500 and mtry at its default value was employed to rank the importance of these 17 variables, with variables exceeding an importance threshold of 2 considered as characteristic variables. The candidate hub genes were then derived by intersecting the characteristic genes identified through these two distinct methods.

### 2.5. Functional Enrichment Analysis

Functional enrichment analysis was conducted utilizing the “clusterProfiler” package, encompassing Gene Ontology (GO) and Kyoto Encyclopedia of Genes and Genomes (KEGG) analyses. Specifically, GO analysis involved the exploration of biological process (BP), cell component (CC), and molecular function (MF). The input genes for this analysis comprised either the DEGs or major module genes identified by the MCODE app, with a significance threshold set at *p* < 0.05.

For the two hub genes obtained through CytoHubba and machine learning methods, aimed at elucidating their potential roles in HBV‐related ALF, we categorized them into high and low expression groups based on the median of their expression levels. Subsequently, Gene set enrichment analysis (GSEA) and gene set variation analysis (GSVA) were performed utilizing the “clusterProfiler,” “GSEABase,” and “GSVA” packages. The KEGG gene sets, comprising 186 biological pathways, along with 50 HALLMARK gene sets, were procured from the MSigDB website (https://www.gsea-msigdb.org/gsea/msigdb) to facilitate these analyses.

### 2.6. Construction of the Nomogram

Utilizing the expression levels of the two identified hub genes, a nomogram was developed to gauge their respective significance and forecast the probability of ALF. Subsequently, a calibration plot was employed to evaluate the alignment between the predicted probability and the actual probability of ALF, assessing the consistency of the predictive model.

### 2.7. Immune Infiltration Analysis

Leveraging the consolidated expression matrix, we assessed immune cell infiltrations in both normal and ALF samples, primarily employing the CIBERSORT and ssGSEA algorithms. The CIBERSORT method facilitated the evaluation of the relative proportions of 22 distinct immune cell types [20], with the exclusion of samples having a *p* value > 0.05. Immune‐related gene sets, encompassing 23 types of immune cells, were curated from published studies [21]. Additionally, the ssGSEA method was employed to compute the relative extent of immune cell infiltrations.

### 2.8. Transcriptional and Posttranscriptional Analysis

Given the conceivable regulatory interplay among transcription factors (TFs), microRNAs (miRNAs), and genes, we conducted predictions for miRNAs and TFs associated with TIMP1 and IL18, utilizing multiple databases and visualizing the outcomes through Cytoscape. To anticipate miRNAs regulating the hub genes, we employed miRWalk, miRDB, and mirDIP databases. For TF predictions, ChEA3 and hTFtarget databases were employed. All parameter settings in the above databases are default parameters. The identification of common miRNAs or TFs resulting from these predictions was facilitated by a Venn diagram.

### 2.9. Statistical Analysis

Data processing and analysis were conducted using R software and online network tools. The comparison of continuous variables between the two groups utilized the Wilcoxon test. Receiver operating characteristic (ROC) analysis was employed to compute the area under the curve (AUC), assessing the diagnostic efficacy of hub genes or the nomogram. The relationships between immune cells and hub genes were evaluated using the Spearman method. A significance level of *p* < 0.05 was adopted as the criterion for determining statistically significant differences.

## 3. Results

### 3.1. Identification of the DEGs

**FIGURE 1:**
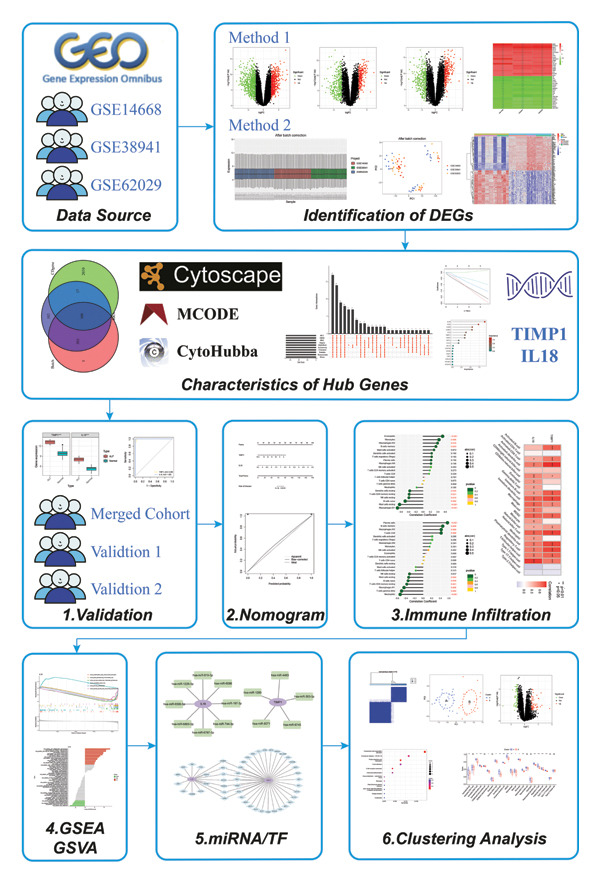
Flow diagram of the study design.

The flowchart was shown in Figure 1. Following standardized processing across all datasets, we initiated a differential expression analysis within each dataset. Employing the specified screening criteria, we identified 642 upregulated and 669 downregulated genes in the GSE14668 dataset, showcasing the top 50 genes with the highest fold change between normal and ALF tissues through a heatmap (Figure 2(a)). Similarly, the GSE38941 dataset revealed 558 DEGs, comprising 237 upregulated and 321 downregulated genes (Figure 2(b)). In the GSE62029 dataset, we pinpointed 575 DEGs, with 255 genes exhibiting elevated expression and 320 genes showing decreased expression in ALF tissues (Figure 2(c)). The “aggregateRanks” function was employed to amalgamate the common DEGs and their respective fold change rankings. Consequently, we identified 319 upregulated genes and 413 downregulated genes, presenting the top 20 genes with the most significant fold changes through a heatmap (Figure 2(d)). Furthermore, the three datasets were amalgamated for subsequent analyses. Boxplots and PCA plots, both before and after correction, demonstrated that sample expressions in different datasets exhibited uniformity, resulting in a more homogeneous sample distribution (Figures 2(e), 2(f)). The integrated dataset results indicated 354 highly expressed genes and 259 lowly expressed genes in ALF tissues (Figure 2(g)).

FIGURE 2Identification of DEGs in HBV–ALF. The volcano and heatmap of DEGs between 8 HBV–ALF and 20 normal liver tissues in GSE14668 (a), 17 HBV–ALF and 10 normal liver tissues in GSE38941 (b), and 13 HBV–ALF and 17 normal liver tissues in GSE62029 (c). (d) Heatmap of the top 20 upregulated and downregulated DEGs using the RRA method. (e) Box plots before and after batch effect correction. (f) PCA plots before and after batch effect correction. (g) Heatmap of the top 50 upregulated and downregulated DEGs using the limma method in the combined cohorts. DEGs, differentially expressed genes; ALF, acute liver failure; RRA, robust rank aggregation; PCA, principal components analysis.

**Figure:**
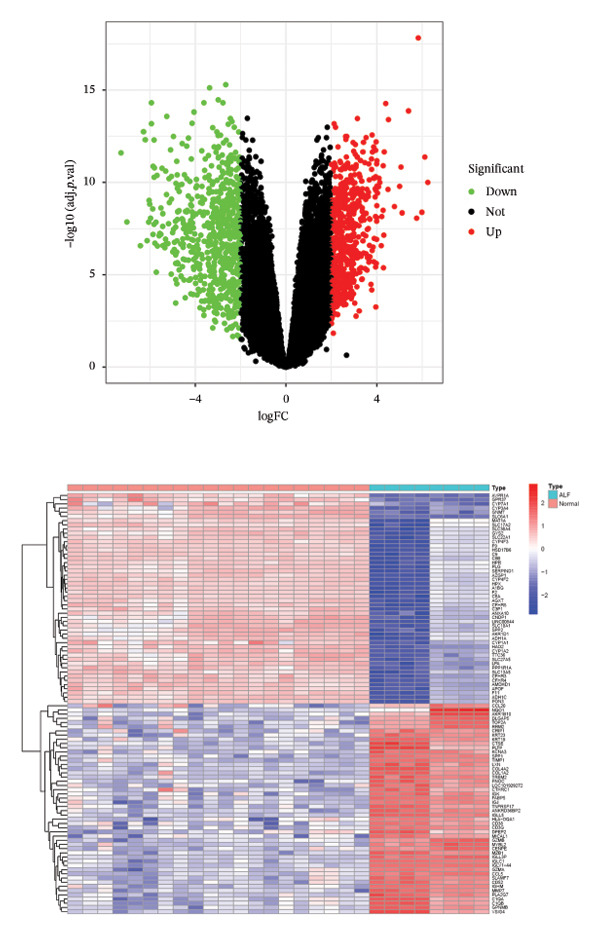
(a)

**Figure:**
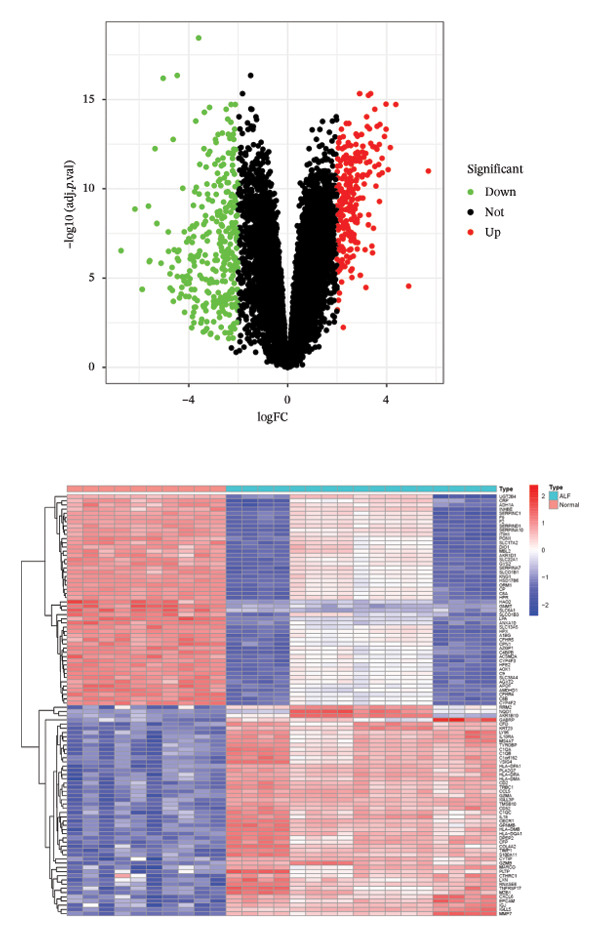
(b)

**Figure:**
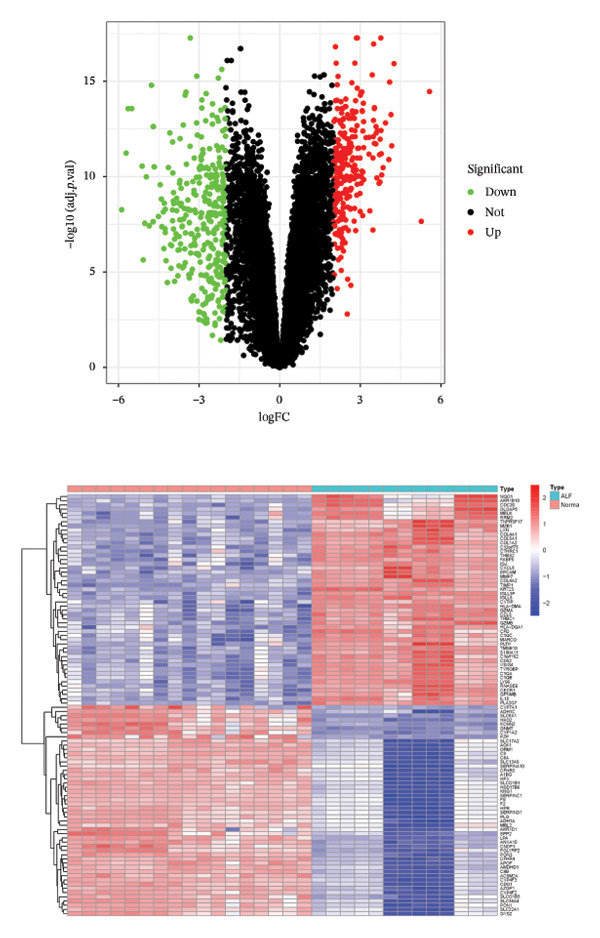
(c)

**Figure:**
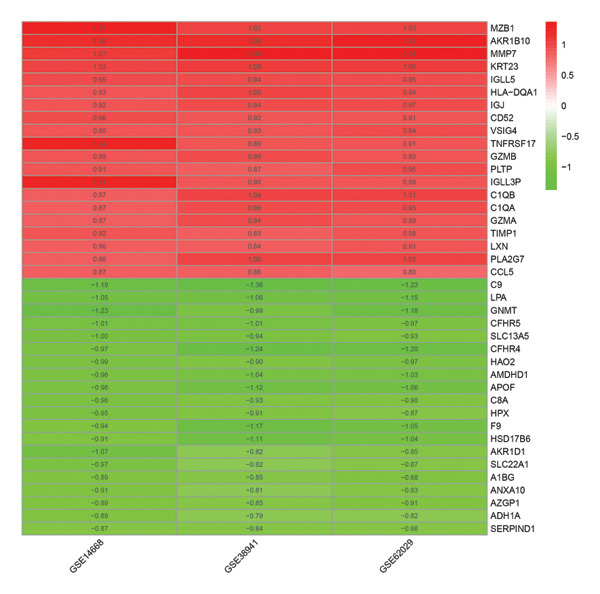
(d)

**Figure:**
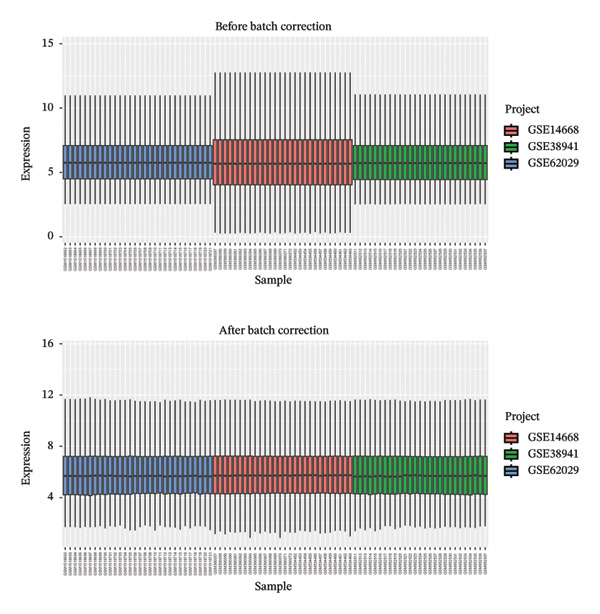
(e)

**Figure:**
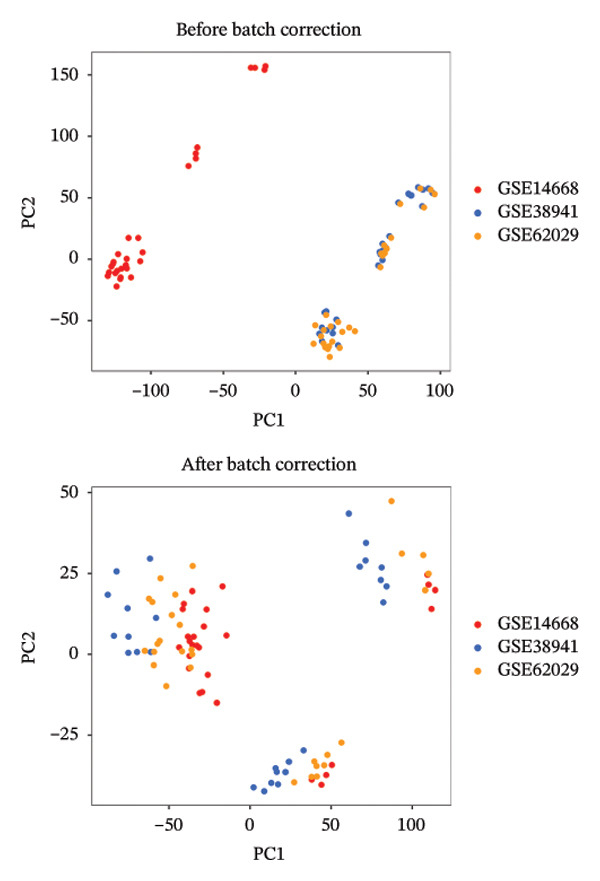
(f)

**Figure:**
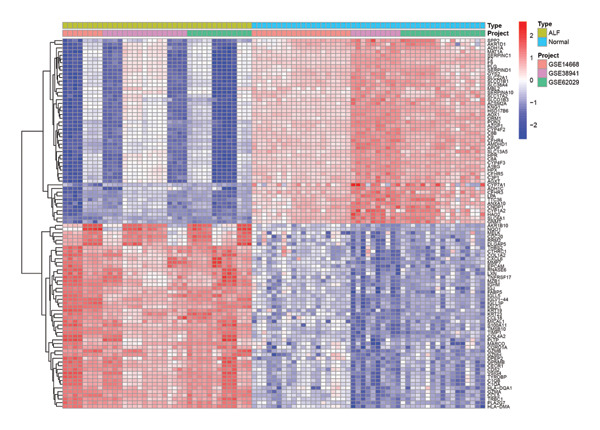
(g)

### 3.2. Analysis of the Network of the PCD Genes

Leveraging the PCD genes and employing the ssGSEA algorithm, we computed scores for various modes of cell death across the integrated samples. The results illuminated substantial disparities in death scores between normal and ALF tissues, manifesting as higher death scores in ALF tissues, except for cuproptosis, ferroptosis, and parthanatos (Figure 3(a)). Subsequently, we intersected the identified DEGs and PCD genes, culminating in the identification of 109 differentially expressed PCD genes (Figure 3(b)). A comprehensive network analysis was conducted for the 109‐gene network, encompassing PPI analysis, enrichment analysis, and module identification. The PPI network analysis revealed close interactions among the proteins encoded by the 109 genes (Figure 3(c)). Enrichment analysis shed light on the functional roles of the network, with GO analysis indicating significant enrichment in BP such as wound healing, regulation of coagulation or hemostasis, and regulation of inflammatory response. In terms of CC, these genes were prominently associated with the collagen‐containing extracellular matrix, endoplasmic reticulum lumen, and vesicle lumen, among others. In terms of MF, the genes were linked to receptor ligand activity or enzyme activity (Figure 3(d)). Additionally, KEGG analysis showcased significant enrichment in fundamental liver functions, including complement and coagulation cascades, lipid and atherosclerosis, hematopoietic cell lineage, platelet activation, vitamin digestion, and absorption. Immune‐related pathways, such as viral protein interaction with cytokine and cytokine receptor, the Toll‐like receptor (TLR) signaling pathway, cytokine–cytokine receptor interaction, and the intestinal immune network for IgA production, were also prominent (Figure 3(e)). Furthermore, utilizing the MCODE algorithm, we identified two modules. Module 1, comprising 16 nodes and 228 edges, is primarily associated with liver metabolism, including fat digestion and absorption, cholesterol metabolism, and vitamin digestion and absorption (Figures 3(f), 3(h)). Module 2, with 26 nodes and 286 edges, predominantly engaged in immune processes such as cytokine–cytokine receptor interaction and chemokine signaling pathway (Figures 3(g), 3(i)). The specific functions of these modules were further elucidated, with Module 1 emphasizing liver metabolism (Figure 3(h)) and Module 2 emphasizing immune processes (Figure 3(i)).

FIGURE 3Enrichment analysis and module identification. (a) Comparison of 15 PCD scores calculated by the ssGSEA method between ALF and normal liver tissues. (b) Identification of 109 differentially expressed PCD genes in ALF through RRA, batch‐diff limma, and PCD genes. (c) Construction of the PPI network using the 109 genes through the STRING database. (d) Bubble plot of GO enrichment results of the 109 genes, including BP, CC, and MF. (e) Bar plot of KEGG enrichment results of the 109 genes. Module 1 (f) and Module 2 (g) were obtained from the PPI network using the MCODE plug‐in. KEGG enrichment results using the genes in Module 1 (h) and Module 2 (i). PCD, programmed cell death; PPI, protein–protein interaction; GO, gene ontology; BP, biological process; CC, cell component; MF, molecular function; KEGG, Kyoto Encyclopedia of Genes and Genomes.

**Figure:**
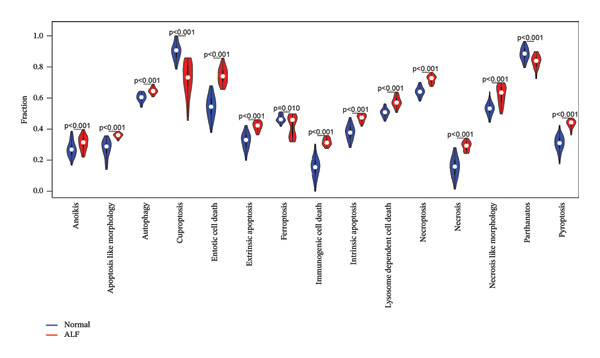
(a)

**Figure:**
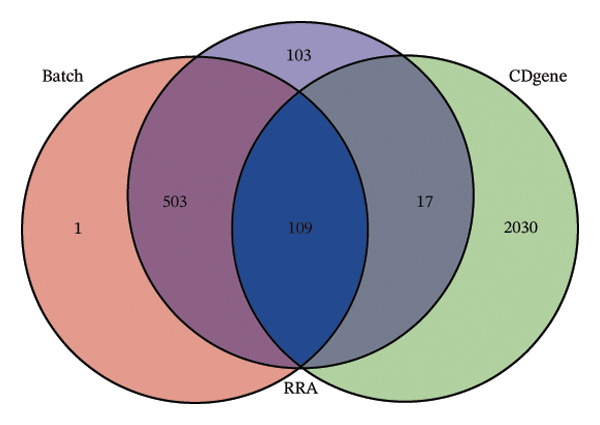
(b)

**Figure:**
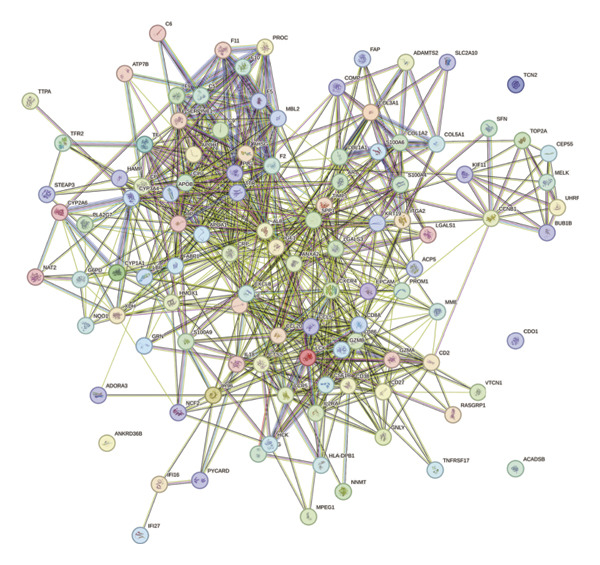
(c)

**Figure:**
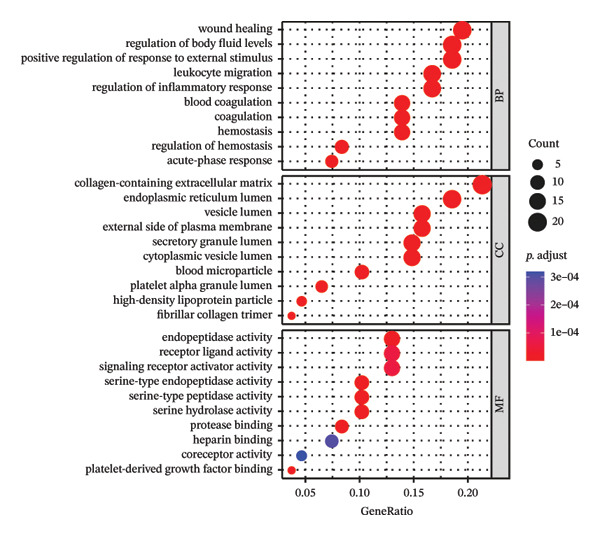
(d)

**Figure:**
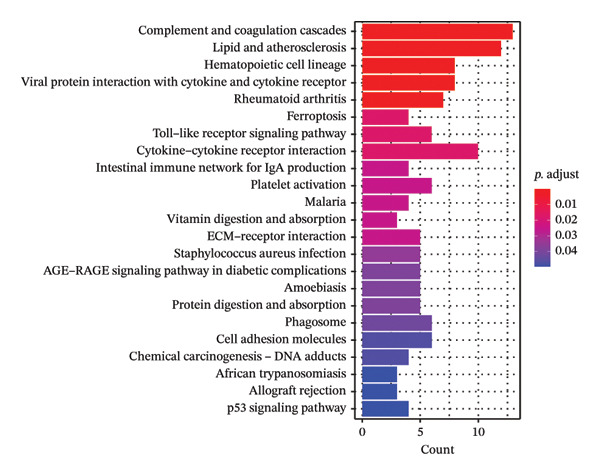
(e)

**Figure:**
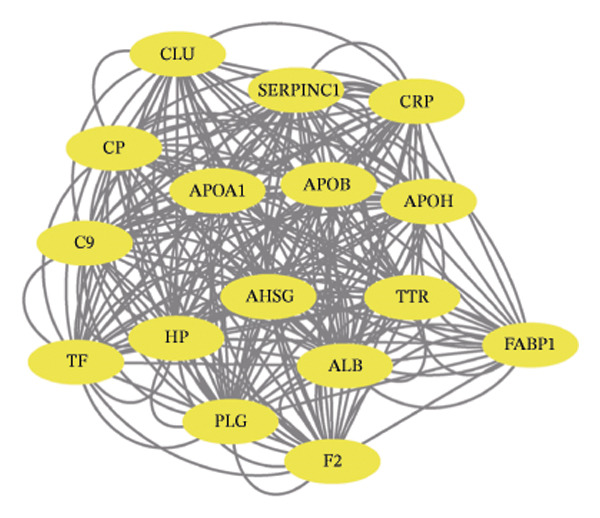
(f)

**Figure:**
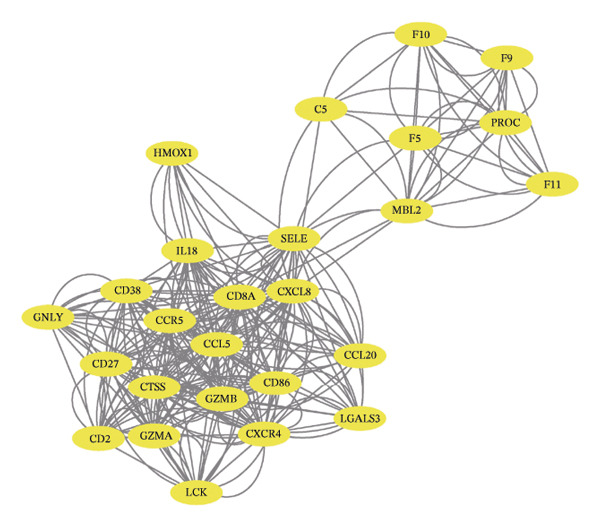
(g)

**Figure:**
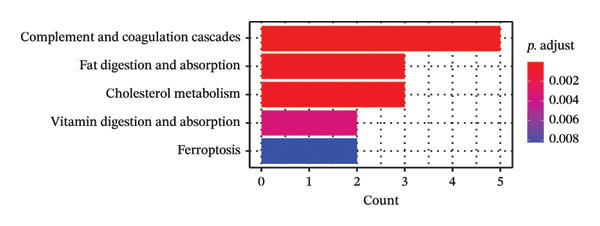
(h)

**Figure:**
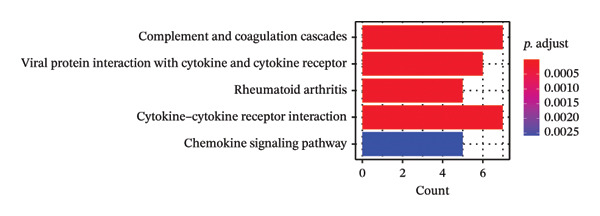
(i)

### 3.3. Identification of the Hub Genes and Construction of the Nomogram

Employing the CytoHubba plug‐in and its suite of 10 algorithms, we computed the top 70 hub genes, ultimately deriving 17 key candidate genes by taking their intersection (Figure 4(a)). In order to comprehensively comprehend the diagnostic potential of these 17 PCD genes, we constructed diagnostic prediction models using two distinct algorithms to discriminate between ALF patients and normal controls. Initially, the LASSO algorithm was employed to select the five ALF‐related characteristics, namely TIMP1, IL18, CXCL10, CCL5, and STAT1 (Figure 4(b)). Subsequently, the RF algorithm was applied to identify feature importance, ultimately choosing the top seven genes with an importance score exceeding 2 as diagnostic genes, namely IL18, CD2, CD86, GZMA, LCK, TIMP1, and CD8A (Figure 4(c)). Finally, we recognized two hub genes, TIMP1 and IL18, for subsequent investigation following the intersection of candidate genes acquired from the LASSO and RF algorithms (Figure 4(d)).

FIGURE 4Acquisition and validation of the hub genes. (a) Upset plot of the 17 genes using ten algorithms (MCC, DMNC, MNC, Degree, BottleNeck, Eccentricity, Closeness, Radiality, Betweenness, and Stress) of the CytoHubba plug‐in. (b) Identification of the key genes among the 17 genes using LASSO regression with the “glmnet” package. (c) Identification of the key genes with importance > 2 among the 17 genes using RF. (d) Two hub genes, TIMP1 and IL18, were obtained by intersection of LASSO and RF results. Verification of the differential expression and diagnostic value of TIMP1 and IL18 using boxplots and ROC curves in the training set (e), GSE120652 and GSE74000 cohorts (f), and GSE168048 ACLF dataset (g). (h) Construction of the nomogram based on the expression of TIMP1 and IL18. (i) Predictive ability of the nomogram through the calibration curve. (j) ROC curve of the nomogram in the diagnosis of ALF. LASSO, least absolute shrinkage and selection operator; RF, random forest; ROC, receiver operating characteristic. ^∗^ *p* < 0.05, ^∗∗^ *p* < 0.01; ^∗∗∗^ *p* < 0.001.

**Figure:**
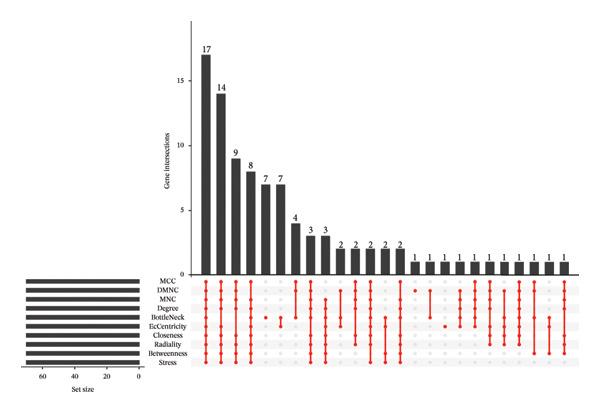
(a)

**Figure:**
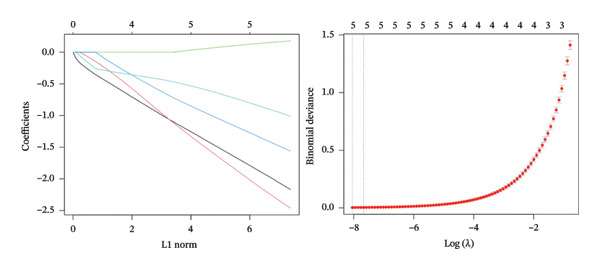
(b)

**Figure:**
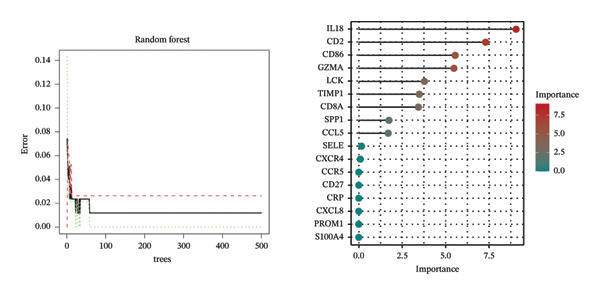
(c)

**Figure:**
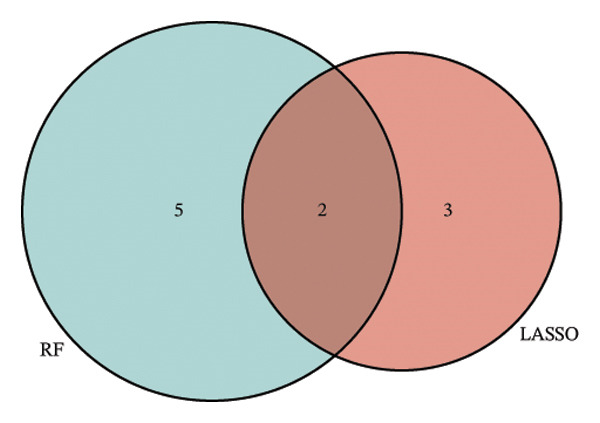
(d)

**Figure:**
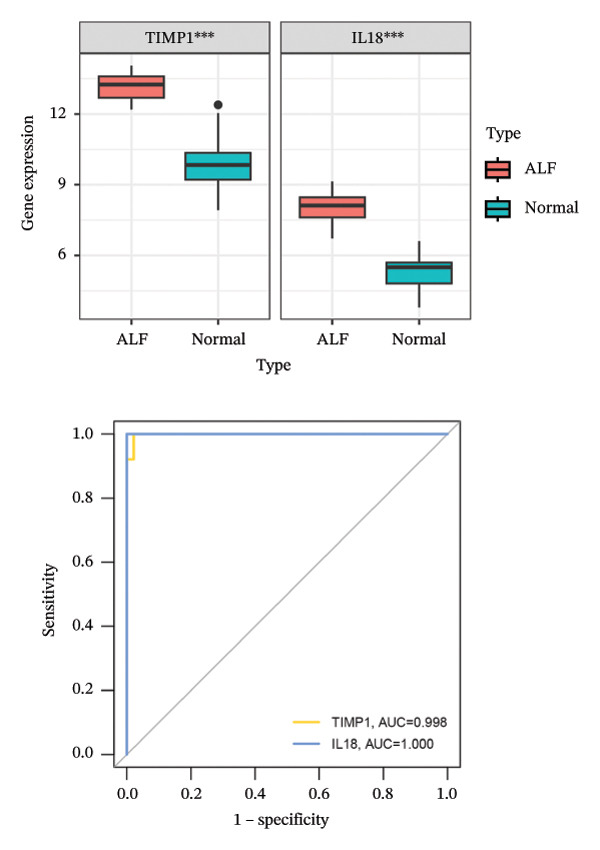
(e)

**Figure:**
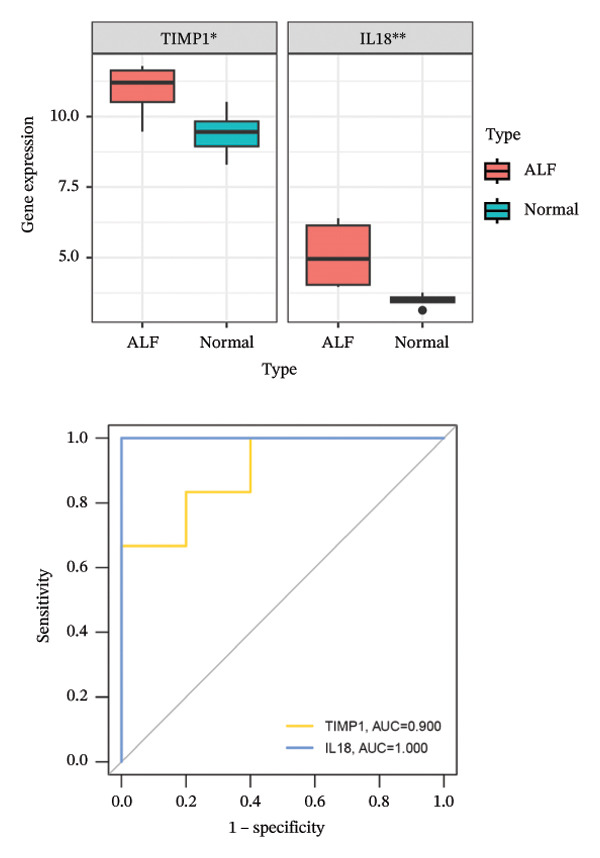
(f)

**Figure:**
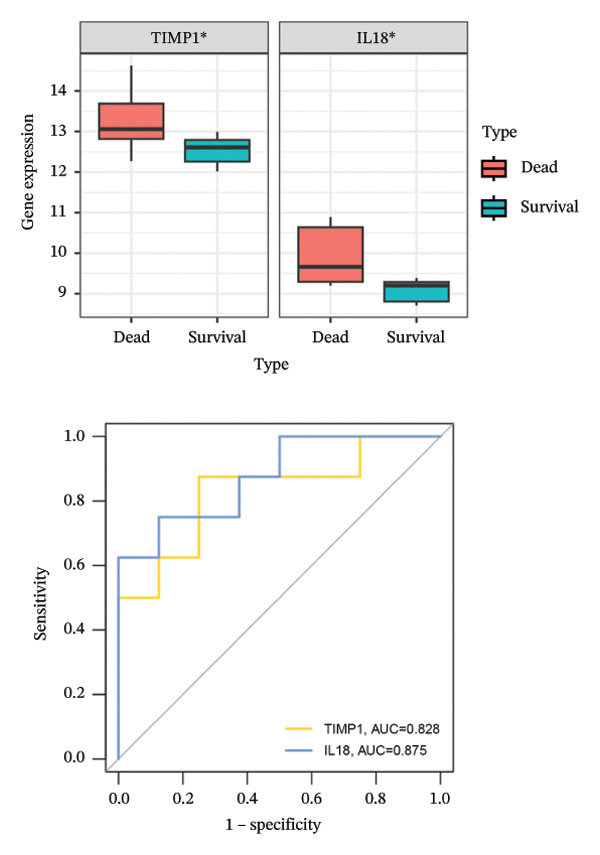
(g)

**Figure:**
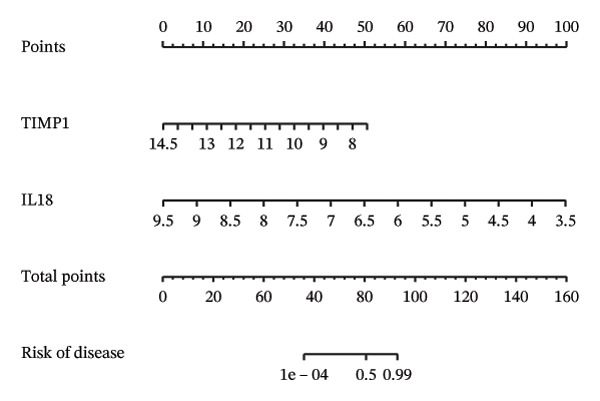
(h)

**Figure:**
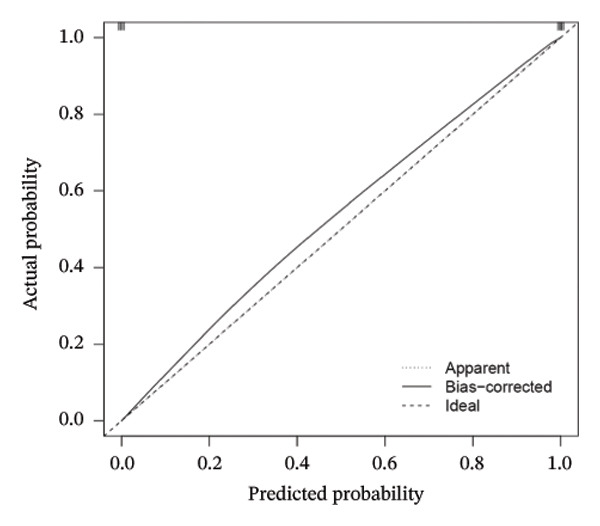
(i)

**Figure:**
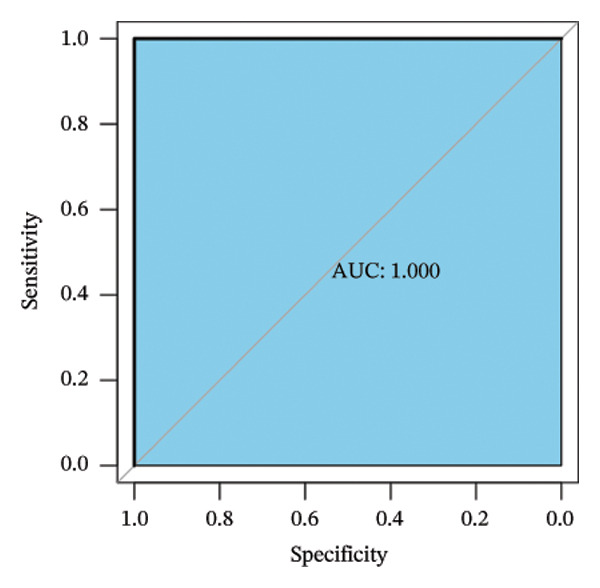
(j)

To assess the diagnostic efficacy of the identified hub genes, we conducted a comprehensive analysis utilizing boxplots and ROC curves in both the training and validation sets. In the training set, both TIMP1 and IL18 exhibited significant overexpression in the ALF group compared to the normal group. The ROC curves demonstrated exceptional diagnostic value, with AUC values of 0.998 for TIMP1 and 1 for IL18 (Figure 4(e)). Following normalization and merging, GSE120652 and GSE74000 were employed as validation sets. Boxplots illustrated significantly higher expressions of TIMP1 and IL18 in ALF groups than in normal controls. The ROC curves reinforced the robust diagnostic performance of TIMP1 and IL18, yielding AUCs of 0.9 and 1, respectively (Figure 4(f)). Additionally, in the ACLF cohort (GSE168048), we explored the predictive potential of these two genes for 28‐day mortality. Results showed higher expressions of TIMP1 and IL18 in ACLF patients who succumbed, with AUCs of 0.828 and 0.875, respectively. This suggested their predictive value for mortality within 28 days among ACLF patients (Figure 4(g)).

Furthermore, we constructed a diagnostic model for ALF utilizing a nomogram that incorporated both TIMP1 and IL18, visually highlighting their respective contributions (Figure 4(h)). The calibration curve underscored the model’s high consistency in predicting the likelihood of ALF (Figure 4(i)). The ROC curve further affirmed the model’s efficacy, yielding an AUC of 1, indicative of its high diagnostic value for ALF (Figure 4(j)).

### 3.4. Evaluation of the Immune Cell Infiltrations

Initially, we assessed the immune infiltration fraction of 22 distinct immune cells in the samples, utilizing the CIBERSORT method, to delineate differences in the immune microenvironment between ALF and normal tissues (Figure 5(a)). ALF patients exhibited elevated infiltration of plasma cells, T cells CD8, T cells CD4 memory activated, and macrophages M0, while experiencing decreased infiltration of T cells CD4 memory resting, T cells follicular helper, macrophages M1, mast cells activated, and neutrophils (Figure 5(b)). Furthermore, we observed that TIMP1 expression was positively correlated with plasma cells, B cells memory, macrophages M2, T cells CD8, and negatively correlated with mast cells resting, B cells naïve, T cells CD4 memory resting, macrophages M1, T cells gamma delta, and neutrophils (Figure 5(c)). IL18 exhibited a positive relationship with eosinophils, monocytes, macrophages M2, B cells memory, and a negative correlation with dendritic cells resting, T cells CD4 memory resting, NK cells resting, B cells naïve, mast cells resting, and macrophages M1 (Figure 5(d)).

FIGURE 5Immune infiltration associated with ALF and hub genes. (a) Stacked bar chart of the relative proportion of 22 immune cells in normal and ALF samples using CIBERSORT. (b) Comparison of the fraction of 22 immune cells between normal and ALF samples using the Wilcoxon test. The relationship between TIMP1 (c), IL18 (d), and immune cell infiltration in ALF samples using the Spearman method. (e) Heatmap of the distribution of 23 immune cells in normal and ALF samples using the ssGSEA method. (f) Violin plot of the distribution of 23 immune cells using the Wilcoxon test. (g) The relationship between the hub genes and immune cell infiltration using the Spearman method. ^∗^ *p* < 0.05, ^∗∗^ *p* < 0.01; ^∗∗∗^ *p* < 0.001.

**Figure:**
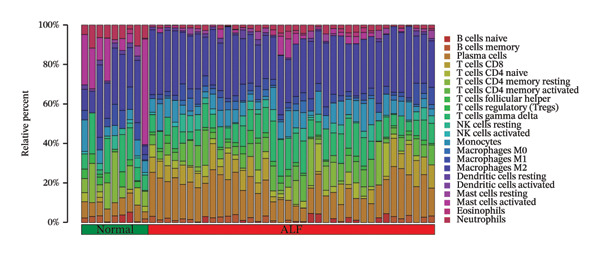
(a)

**Figure:**
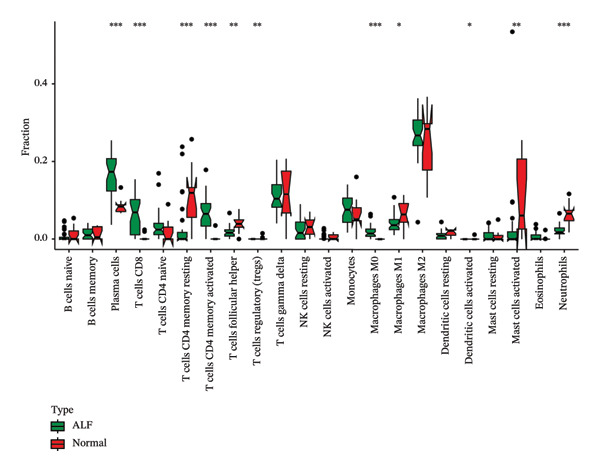
(b)

**Figure:**
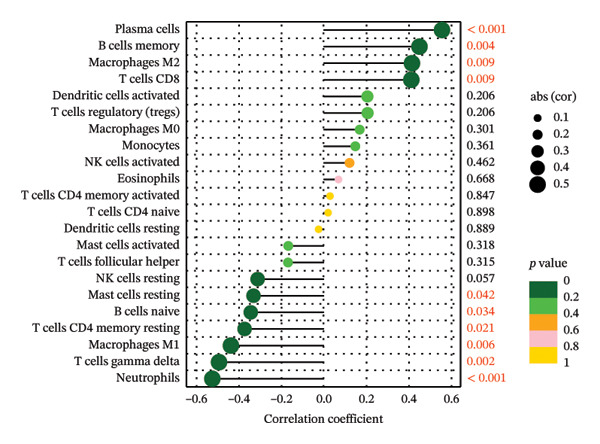
(c)

**Figure:**
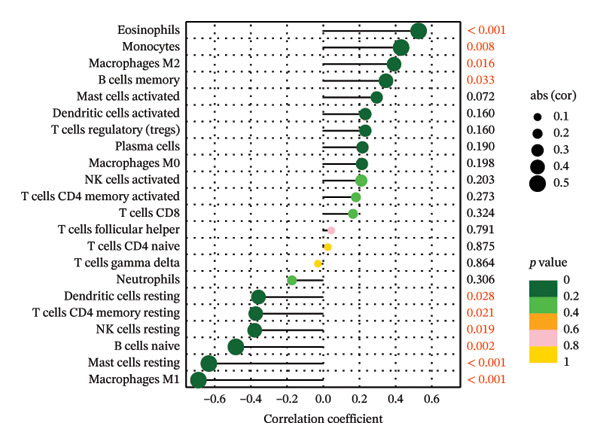
(d)

**Figure:**
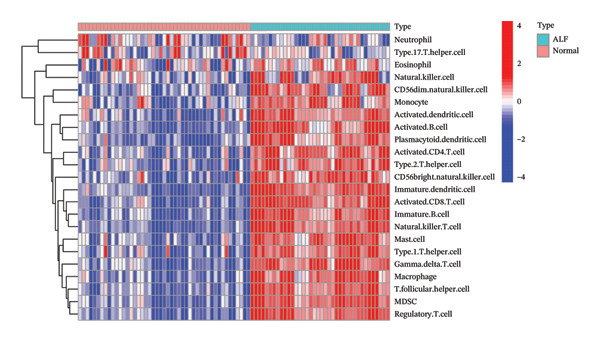
(e)

**Figure:**
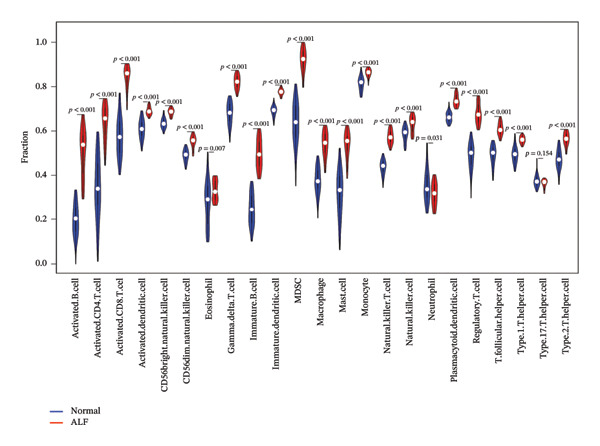
(f)

**Figure:**
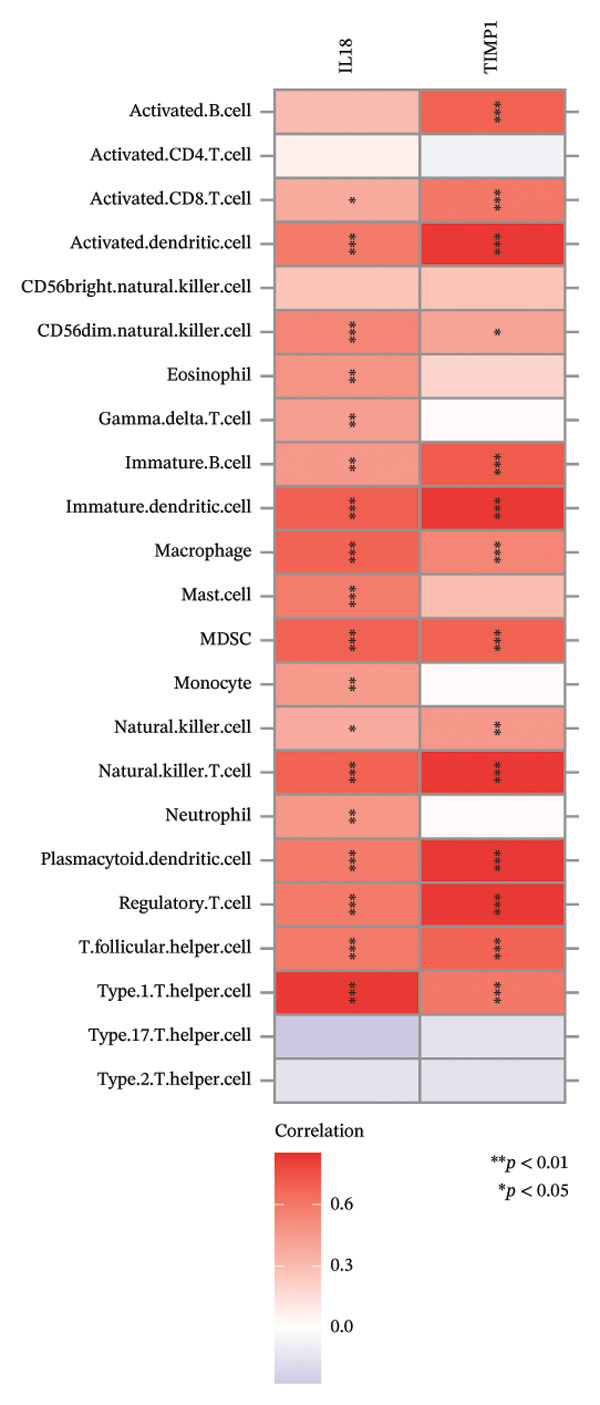
(g)

Subsequently, immune‐cell infiltration was reassessed using the ssGSEA algorithm. The results demonstrated significantly heightened infiltration of nearly all immune cells in ALF samples compared to normal samples, except for a lower infiltration of neutrophils (Figures 5(e), 5(f)). Correlation analysis further revealed a positive association between TIMP1 and IL18 and most immune cells (Figure 5(g)). In summary, the expression levels of TIMP1 and IL18 are significantly correlated with the infiltration degree of specific immune cells.

### 3.5. Functional Analysis of TIMP1 and IL18

To delve deeper into the molecular mechanisms underpinning the diagnostic biomarkers for ALF, GSEA was conducted for each gene biomarker. The pathways enriched in low expression of TIMP1 and IL18 encompassed complement and coagulation cascades, along with metabolism‐related pathways such as drug metabolism cytochrome p450 and retinol metabolism, among others (Figures 6(a), 6(b), Table S1, Table S2). Notably, the IL18 high expression group was associated with the leishmania infection pathway (Figure 6(b)). Upon categorizing ALF samples into high and low expression groups based on the median expression of the two biomarkers, GSVA unveiled distinct pathways between the groups. The comprehensive analysis suggested that high expression of TIMP1 or IL18 may be positively related to the pathways associated with allograft rejection, inflammatory response, myogenesis, angiogenesis, apoptosis, and classic signaling pathways (EMT, PI3K_AKT_MTOR, IL6_JAK_STAT3, and IL2_STAT5). Conversely, low expression of TIMP1 or IL18 appeared to be associated with pathways related to cholesterol homeostasis, heme metabolism, oxidative phosphorylation, and DNA repair, among others (Figures 6(c), 6(d)).

FIGURE 6Biological function of the two hub genes. GSEA analysis of TIMP1 (a) and IL18 (b) using KEGG gene sets. GSVA analysis of TIMP1 (c) and IL18 (d) using HALLMARK gene sets. GSEA, gene set enrichment analysis; GSVA, gene set variation analysis.

**Figure:**
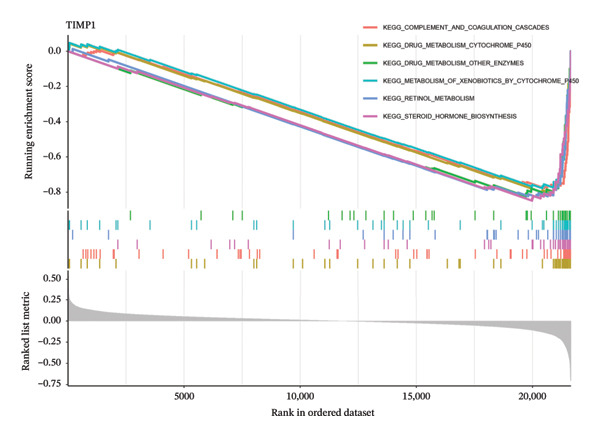
(a)

**Figure:**
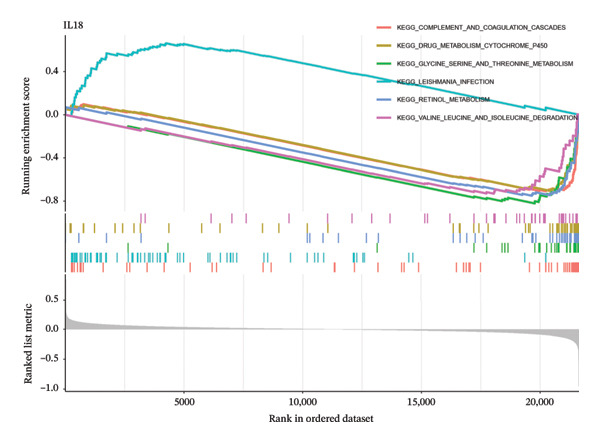
(b)

**Figure:**
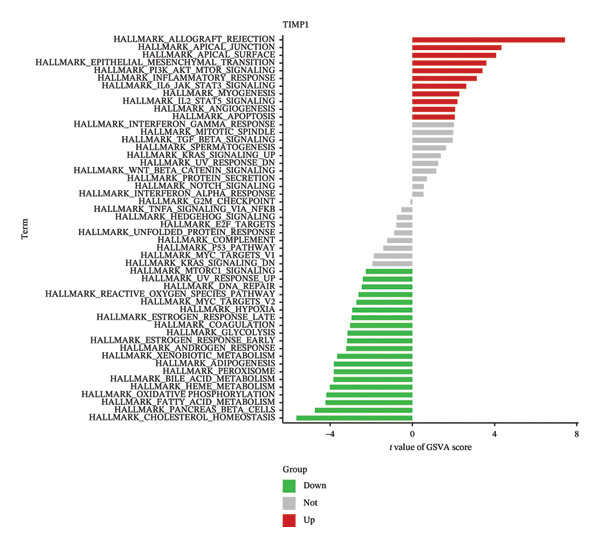
(c)

**Figure:**
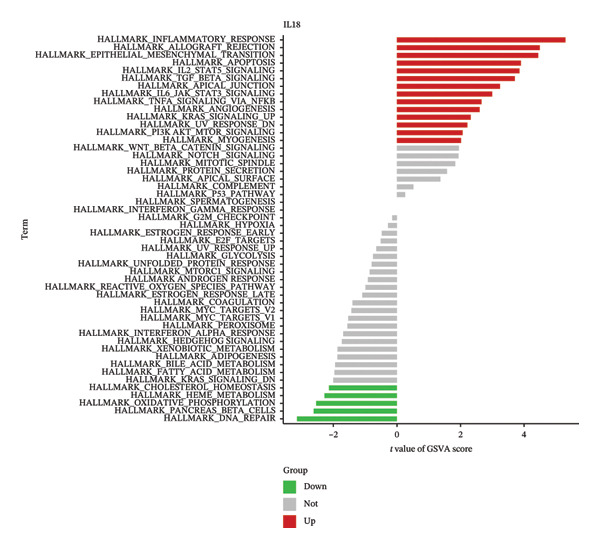
(d)

### 3.6. Construction of the miRNA–TF–mRNA Regulatory Network

Utilizing three databases (miRWalk, mirDIP, and mirRDB), we conducted an exploration to predict miRNAs with potential binding to the two identified hub genes. Our analysis revealed a total of 8 miRNAs binding to IL18 and 5 miRNAs binding to TIMP1 (Figure 7(a)), suggesting an intricate network contributing to the regulation of hub gene expression. Subsequently, the miRNA–mRNA regulatory network, comprising two hub genes and 13 miRNAs, was delineated and visually presented using Cytoscape software (Figure 7(b)). Additionally, predictions of TFs binding to the two genes were made using the ChEA3 and hTFtarget databases. A total of 17 TFs were identified as regulators of IL18, while 38 TFs were implicated in the regulation of TIMP1 (Figure 7(c)). The TF‐gene regulatory network encompassed 50 nodes and 55 edges, revealing 7 TFs (CEBPB, MYC, JUN, STAT3, TFAP2C, FOS, and JUND) with shared regulatory influence on both genes (Figure 7(d)). We further scrutinized the differential expression of these 7 TFs between normal and ALF tissues and among survival and deceased patients in the training cohort and two validation cohorts. The outcomes indicated that only STAT3 exhibited differential expression across all three cohorts, with lower expression in ALF tissues and survival patients. Simultaneously, its AUC values in the three cohorts were 0.887, 0.967, and 0.891, respectively, signifying high diagnostic value. These bioinformatics analysis results suggest that STAT3 may be a potential upstream factor regulating these two key genes, but its specific regulatory mechanism and causal relationship in ALF require further experimental studies for confirmation (Figures 7(e), 7(f), 7(g)).

FIGURE 7Regulatory network of the two hub genes. (a) Prediction of miRNA regulating IL18 and TIMP1 using miRDB, miRWalk, and mirDIP databases. (b) miRNA–mRNA network visualized by Cytoscape. (c) Prediction of TF regulating IL18 and TIMP1 using ChEA3 and hTFtarget databases. (d) TF–mRNA network visualized by Cytoscape. Verification of the differential expression and diagnostic value of the seven common TF using boxplots (Wilcoxon test) and ROC curves in the training set (e), GSE120652 and GSE74000 cohorts (f), and GSE168048 ACLF dataset (g). TF, transcription factor. ^∗^ *p* < 0.05, ^∗∗^ *p* < 0.01; ^∗∗∗^ *p* < 0.001.

**Figure:**
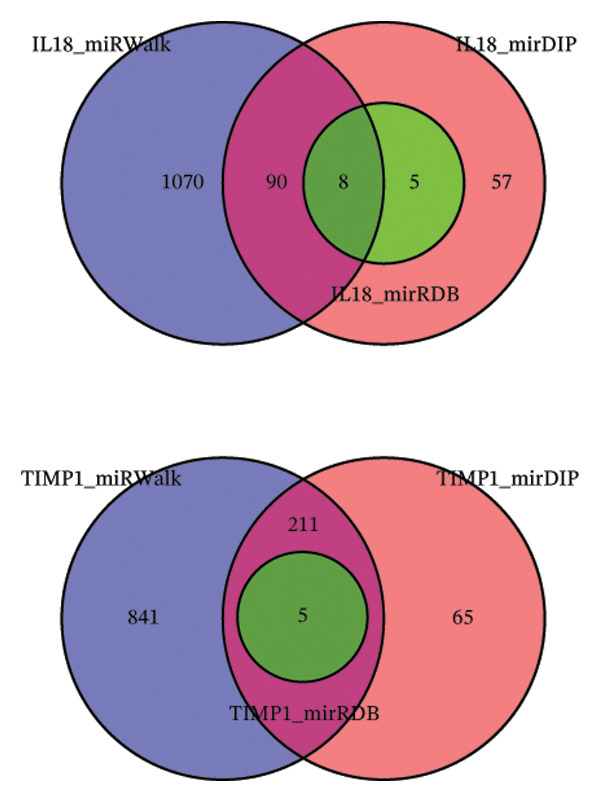
(a)

**Figure:**
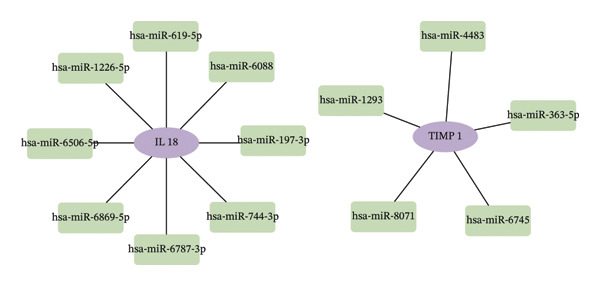
(b)

**Figure:**
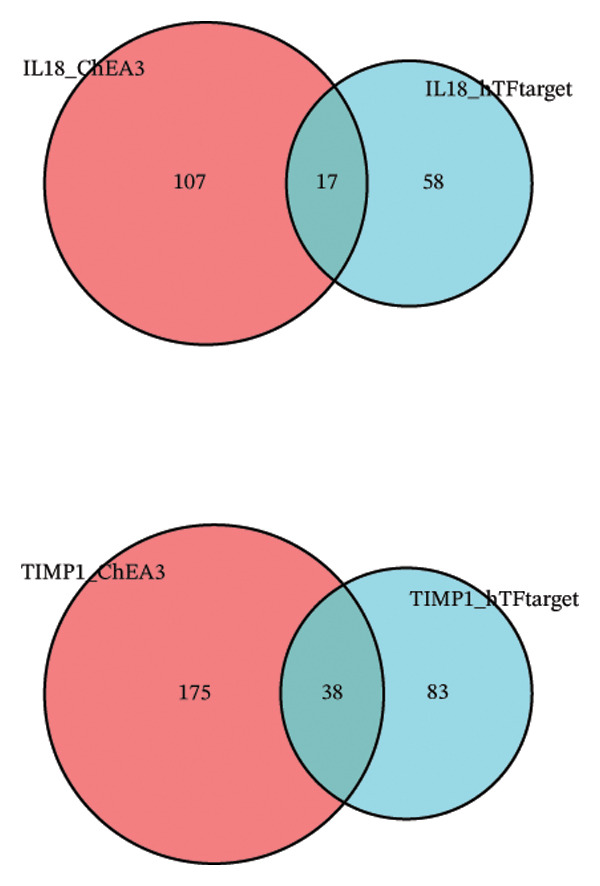
(c)

**Figure:**
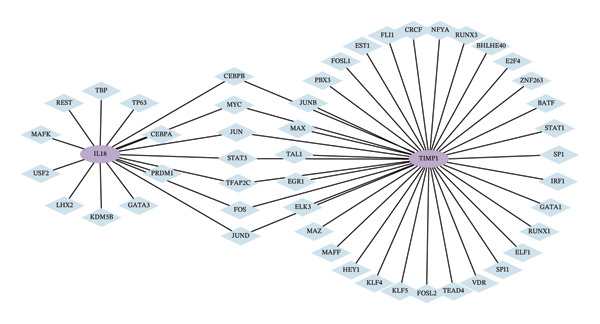
(d)

**Figure:**
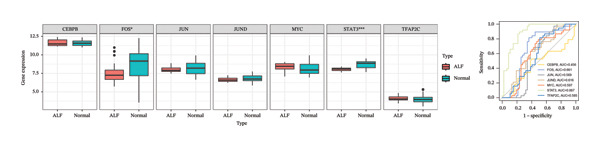
(e)

**Figure:**
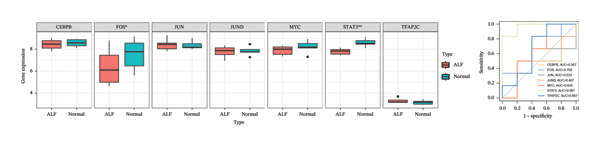
(f)

**Figure:**
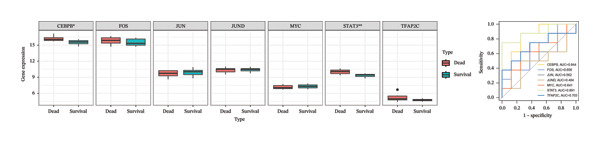
(g)

## 4. Discussion

Numerous studies have extensively investigated PCD in various tumor diseases, such as liver cancer, prostate cancer, and melanoma, among others [22–25]. Despite the wealth of research in this area, there remains a notable gap in the literature regarding a comprehensive analysis of the significance of PCD in ALF. Given the foundational pathological process of ALF involving extensive hepatocyte death, our study represents the inaugural exploration into the relevant role of PCD and the identification of pivotal PCD genes in ALF. Employing the ssGSEA method, we estimated PCD scores in both normal and ALF tissues, revealing higher scores across most death processes in ALF tissues [26]. This underscores a crucial connection between PCD and ALF. Previous investigations have highlighted the presence of apoptosis, necrosis, autophagy, and ferroptosis in the progression of ALF. Consequently, leveraging the GEO database, we embarked on a comprehensive analysis, encompassing the identification of hub genes, the evaluation of their significance in ALF through nomogram construction, correlation with immune infiltration, elucidation of biological functions, and construction of regulatory networks.

In this investigation, we initiated the identification of DEGs in ALF by employing the RRA method and the “batch diff” method. Subsequent intersection with previously recognized PCD genes yielded a pool of 109 PCD genes exhibiting aberrant expression patterns in ALF. Enrichment analysis unveiled that these DEGs predominantly participated in essential physiological functions and immune‐related pathways within the liver. The enriched pathways encompassed vital liver functions such as complement and coagulation cascades, lipid metabolism, platelet activation, and digestion and absorption of vitamins, fats, and proteins. The liver, as the primary site for synthesizing various coagulation factors, serves as the metabolic hub for sugars, proteins, fats, vitamins, and hormones. Both complement and coagulation systems, activated by pathway‐specific triggers, share a common evolutionary origin. Interactions between these homologous systems can influence their activation, amplification, and regulation, emphasizing their intertwined nature [27, 28]. Particularly, the complement and coagulation cascade pathways have been implicated in the pathogenesis of HBV–ALF [29]. Moreover, our findings align with the understanding that the systemic inflammatory response plays a crucial role in ALF progression [30, 31]. Previous studies have elucidated key immunological mechanisms in ALF, encompassing acquired neutrophil dysfunction, TLR function, and the significant impact of chemokine and cytokine storms [32–36]. Cytokines, pivotal in hepatocyte regeneration and death, are integral to ALF pathophysiology [37, 38]. Consistent with our investigation, the enrichment of chemokines and cytokine‐related pathways underscores their pivotal role in ALF.

In this investigation, we discerned two potential diagnostic genes, TIMP1 and IL18, utilizing a combination of bioinformatics and machine learning approaches. The extremely high discrimination efficiency of TIMP1, IL18, and the nomogram constructed in this study in the training set and independent verification set is encouraging, but it should also be treated with caution. This may reflect the core role of the selected genes in the pathological process of ALF, but it may also be affected by batch effect between the analyzed public datasets, homogeneity of disease subtypes, or relatively limited sample size, with a certain degree of risk of overfitting. Its generalization ability needs to be verified in a larger, multicenter clinical cohort. TIMP1 has been identified as a key participant in hepatocyte apoptosis–fibrosis dynamics. Notably, the upregulation of TIMP1 is associated with hepatocyte apoptosis, and its inducibility is apoptosis‐dependent. Upon inhibition of TIMP1, the liver’s fibrotic response diminishes, accompanied by downregulation of fibrosis‐related genes such as aSMA, CTGF, and TGFb2r [39]. Chen et al. observed a significant elevation of TIMP1 in the central nervous system after unilateral intraventricular injection of TIMP1 cDNA plasmid in ALF mice, leading to reduced blood–brain barrier permeability, decreased activation of EGFR and p38 MAPK, and restoration of the tight junction protein occludin. This underscores the potential therapeutic role of TIMP1 in ALF [40]. Furthermore, TIMP1 emerges as a key gene in ACLF, exhibiting close associations with immune response and metabolic processes. It stands out as a potential target for prognosis prediction and molecular‐targeted therapy in ACLF cases [41]. Noteworthy is its significance in posthepatectomy liver failure, where plasma concentrations of IL18 rise in the later phase after one week. In rat experiments, it was found that IL18 undergoes metabolism in the liver and is excreted in bile, and the heightened plasma IL18 in liver failure patients reflects compromised liver metabolism [42]. In fulminant hepatic failure, both serum and liver tissue levels of IL18 register a significant increase [43]. Among ACLF patients, elevated concentrations of IL18 correlate with increased proportions of NKB cells and higher levels of plasma IL12, both acting as valuable prognostic indicators for the 28‐day survival status. In ACLF, IL18 concentrations exert a positive influence on NKB cells, suggesting a potential positive feedback loop [44]. TIMP1 and IL18 showed the potential to be associated with short‐term mortality in the ACLF cohort, suggesting that they may have a broader prognostic value in severe liver disease associated with acute liver injury, but their exact prognostic significance in simple ALF needs to be confirmed by prospective prognostic studies specifically targeting ALF. Additionally, STAT3, a crucial TF in ALF, has been implicated in the regulation of liver injury through the miRNAs‐124‐mediated targeting of the IL‐6/STAT3 signaling pathway [45]. Exosomes derived from daucosterol, in conjunction with umbilical cord mesenchymal stem cells, exhibit hepatoprotective effects in liver failure mice by modulating the IL‐6/STAT3 signaling pathway [46]. In summary, TIMP1 and IL18 emerge as pivotal players in ALF, offering promise as potential diagnostic markers and candidate therapeutic targets.

The mechanisms underlying ALF encompass both direct liver injury and immune‐mediated liver injury. Wu et al. conducted a comprehensive review revealing the intricate involvement of both innate and adaptive immune systems in immune‐mediated liver injury during ALF. The innate immune response, characterized by swifter activation, exerts a more pronounced impact than adaptive immunity. Furthermore, this immune response interplays with various physiological or pathophysiological factors, including coagulation factors, ultimately determining the outcome of ALF [47]. Emerging evidence suggests a pivotal role for PCD in both innate and adaptive immune responses [48]. PCD’s critical involvement in infiltrating cells within the tumor immune microenvironment raises the hypothesis that PCD might regulate the immune microenvironment, thereby contributing to the onset and progression of ALF. Monocytes and macrophages, vital components of the innate immune system, assume a crucial role in ALF [49]. Kupffer cells (KCs), residing macrophages in the liver, have been investigated by Xie and Ouyang, revealing their exhaustion during the early stages of ALF. Consequently, monocytes are recruited to replace exhausted macrophages, eventually differentiating into mature, activated, and polarized macrophages within the liver’s immune microenvironment. Regulating the balance between M1 and M2 macrophages in ALF emerges as a potential strategy to mitigate liver injury [13]. In the realm of tumor research, M1 macrophages contribute to antitumor immune responses, while M2 macrophages support the survival, proliferation, and metastasis of tumor cells, concurrently inhibiting the function of other immune cells to foster an immunosuppressive response. In our study, we observed diminished infiltration of M1 macrophages alongside elevated levels of M0 macrophages in ALF. Intriguingly, the expression levels of TIMP1 and IL18 exhibited positive correlations with M2 macrophages and negative correlations with M1 macrophages, implying a potential linkage between the identified hub genes and macrophage polarization in the pathogenesis of ALF. However, in the context of ALF, a large number of hepatocyte deaths and a strong inflammatory response may lead to drastic changes in the transcriptome of liver tissue. These algorithms may be interfered with by the specific gene expression of non‐immune cells (such as injured hepatocytes and activated stellate cells). Therefore, the observed changes in the proportion of immune cells should be regarded as an association signal based on gene expression characteristics, and its accuracy needs to be verified at the tissue or cell level by immunohistochemistry, flow cytometry, and other experimental means.

Despite the systematic approach adopted in this study, several limitations warrant consideration. Firstly, our analyses relied on publicly available microarray datasets, which inherently vary in sample collection protocols, clinical covariates, and batch effects, potentially introducing heterogeneity and confounding influences. Secondly, although internal validation was performed using independent GEO cohorts, the absence of external validation with prospectively collected clinical samples limits the immediate translational applicability of TIMP1 and IL18 as biomarkers. The findings of this study belong to the preliminary exploration and discovery stage. The value of TIMP1 and IL18 as biomarkers or targets needs further validation in prospective clinical cohorts (especially based on noninvasive samples such as serum/plasma) and experimental studies in the future. Thirdly, immune infiltration and functional enrichment analyses were conducted in silico using deconvolution algorithms and pathway databases, which, while informative, require experimental verification such as flow cytometry or immunohistochemistry to confirm the cellular and molecular interactions proposed. Fourthly, this study revealed gene expression changes at the tissue level. When translating it into clinical application, we need to explore its expression level in more accessible body fluids, such as peripheral blood, or the concentration change of secreted protein, so as to have practical diagnostic or prognostic monitoring value. Finally, prognostic assessments incorporated data from ACLF patients, which, although relevant, may not fully represent the pathophysiological context of pure ALF. Thus, cautious interpretation is advised when extrapolating these prognostic insights to de novo ALF populations.

In summary, leveraging public datasets, bioinformatics, and machine learning methodologies, we uncovered the significance of PCD in ALF and identified TIMP1 and IL18 as hub genes. Utilizing these hub genes, we formulated a nomogram to predict ALF risk and delved into the essential role of macrophages within the immune microenvironment. Concurrently, we constructed regulatory networks. This study offers a potential therapeutic target for ALF in future investigations.

**Nomenclature**
ACLF: Acute‐on‐chronic liver failure
ALF: Acute liver failure
AUC: Area under the curve
BP: Biological process
CC: Cell component
CDF: Cumulative distribution function
DEGs: Differentially expressed genes
GO: Gene ontology
GSEA: Gene set enrichment analysis
GSVA: Gene set variation analysis
HBV: Hepatitis B virus
KCs: Kupffer cells
KEGG: Kyoto Encyclopedia of Genes and Genomes
LASSO: Least absolute shrinkage and selection operator
MF: Molecular function
PCA: Principal component analysis
PCD: Programmed cell death
PPI: Protein–protein interaction
RF: Random forest
ROC: Receiver operating characteristic
RRA: Robust rank aggregation
TFs: Transcription factors
TLR: Toll‐like receptor

## Author Contributions

Chenjie Qiu contributed to the conception and design of the study. Guangli Liu and Huili Wu collected the databases and performed the analysis. Guangli Liu and Wenxiang Shi wrote the first draft of the manuscript. Chenjie Qiu assisted with data analysis and language improvement.

## Funding

The authors have nothing to report.

## Disclosure

All authors read and approved the final manuscript.

## Ethics Statement

The authors have nothing to report.

## Conflicts of Interest

The authors declare no conflicts of interest.

## Supporting Information

Table S1 GSEA results of IL18.

Table S2 GSEA results of TIMP1.

## Supporting information


**Supporting Information** Additional supporting information can be found online in the Supporting Information section.

## Data Availability

The data are available from the corresponding author for reasonable requests.
